# Why Residents Quit: National Rates of and Reasons for Attrition Among Emergency Medicine Physicians in Training

**DOI:** 10.5811/westjem.2018.11.40449

**Published:** 2019-02-11

**Authors:** Dave W. Lu, Nicholas D. Hartman, Jeffrey Druck, Jennifer Mitzman, Tania D. Strout

**Affiliations:** * Tufts University School of Medicine, Department of Emergency Medicine, Medford, Massachusetts; † Maine Medical Center, Department of Emergency Medicine, Portland, Maine; ‡ Wake Forest School of Medicine, Department of Emergency Medicine, Winston-Salem, North Carolina; § The University of Colorado School of Medicine, Department of Emergency Medicine, Aurora, Colorado; ¶ The Ohio State University College of Medicine, Department of Emergency Medicine, Columbus, Ohio

## Abstract

**Introduction:**

Recruiting and retaining residents who will complete their emergency medicine (EM) training is vital, not only because residency positions are a limited and costly resource, but also to prevent the significant disruptions, increased workload, and low morale that may arise when a resident prematurely leaves a program. We investigated national rates of EM resident attrition and examined the reasons and factors associated with their attrition.

**Methods:**

In this retrospective, observational study we used national data from the American Medical Association National Graduate Medical Education Census for all residents who entered Accreditation Council for Graduate Medical Education-accredited EM programs between academic years 2006–2007 and 2015–2016. Our main outcome was the annual national rate of EM resident attrition. Secondary outcomes included the main reason for attrition as well as resident factors associated with attrition.

**Results:**

Compared to the other 10 largest specialties, EM had the lowest rate of attrition (0.8%, 95% confidence interval [CI] [0.7–0.9]), or approximately 51.6 (95% CI [44.7–58.5]) residents per year. In the attrition population, 44.2% of the residents were women, a significantly higher proportion when compared to the proportion of female EM residents overall (38.8%, p=0.011). A greater proportion of Hispanic/Latino (1.8%) residents also left their programs when compared to their White (0.9%) counterparts (p<0.001). In examining reasons for attrition as reported by the program director, female residents were significantly more likely than male residents to leave due to “health/family reasons” (21.5% vs 9.6%, p=0.019).

**Conclusion:**

While the overall rate of attrition among EM residents is low, women and some under-represented minorities in medicine had a higher than expected rate of attrition. Future studies that qualitatively investigate the factors contributing to greater attrition among female and some ethnic minority residents are necessary to inform efforts promoting inclusion and diversity within the specialty.

## INTRODUCTION

Methods of resident selection for graduate medical training have been widely studied, with prior work examining factors used by programs to select residents as well as exploring predictors of resident success during training.^1^,^2^ One driver for the numerous analyses of the residency selection process is that training programs invest a significant amount of effort and resources to recruit and develop successful residents.^3^,^4^ The premature loss of a resident during training for any reason is disruptive and can create significant difficulties for programs in terms of patient care responsibilities, increased burdens on other providers, and program morale.^5^ For emergency medicine (EM), the competition for coveted residency positions has become increasingly intense.^6^

To ensure that this limited resource is allocated justly and effectively, it is incumbent upon programs to enroll those applicants who are likely to successfully complete residency training. Although other specialties have studied the factors surrounding attrition, EM has not investigated how often attrition occurs among its trainees and for what reasons.^7^–^9^ This study’s primary objective was to examine national rates of resident attrition in Accreditation Council for Graduate Medical Education (ACGME)-accredited EM programs between 2006 and 2016. Secondary objectives included investigating the reasons for attrition as well as the resident characteristics associated with attrition.

## METHODS

### Study Design and Setting

This was a retrospective observational study using de-identified complete national data from the annual American Medical Association (AMA) National Graduate Medical Education (GME) Census.

### Study Population

The study population included all categorical residents without prior United States graduate medical education training who entered ACGME-accredited EM programs between academic years 2006–2007 and 2015–2016. The attrition group consisted of any resident at any level who left his or her program during a specific year.

### Measurements

We calculated the attrition rate for any year using aggregated national data as the percentage of all residents who left their programs. To ensure anonymity the de-identified dataset included resident characteristics that were limited to gender, race/ethnicity, and medical school type (i.e., allopathic, osteopathic, and international). Per the census database, a primary status and reason for each resident departure was reported by the program director (PD). Attrition statuses included the following: 1) dismissal; 2) transfer to another EM program; 3) transfer to a non-EM program; 4) transfer unknown; and 5) withdrawn. Transfer “unknown” means whether it was unknown by the PD at the time of the report to the AMA National GME Census to what specialty the trainee transferred. Reasons for attrition included the following: 1) change in career plans; 2) health/family reasons; 3) military obligations; and 4) other/unknown.

There are two main ways to view resident attrition: There is attrition from the training program the resident initially enrolled in, and there is attrition from the specialty altogether. For several reasons, we chose the most inclusive definition by counting all attrition statuses, including attrition from one EM program to go to another EM program as well as attrition from the specialty altogether. First, we wanted to be consistent with prior work in other specialties so as to be able to compare our results.^10^ Second, attrition from a program or a specialty results in the same negative consequences of morale, workload, and scheduling for the program and its remaining residents. Third, residents who leave one EM program to go to another EM program may highlight the unique systemic challenges he or she faced in that particular program, rather than challenges due to a poor specialty choice, which one presumes would result in attrition to another specialty. Since we were unable to parse out specific details of why each resident left his or her program based on the attrition status and reason reported by PDs, we aimed to provide the most inclusive definition of attrition to gain the most complete picture.

### Outcomes

Our primary outcome was the annual national rate of EM resident attrition. Secondary outcomes included the main status and reason for attrition as well as resident characteristics associated with attrition.

### Data Analysis

We analyzed data using SPSS for Windows v24.0 statistical software (SPSS, Inc., Chicago, Illinois). To assess for the presence of differences in attrition as well as the status and reason for attrition based upon resident characteristics (i.e., gender, race/ethnicity, medical school type), we employed chi-square analyses followed by the Marascuilo procedure where appropriate for the data.^11^ To ensure differences in attrition by gender were not due to potentially changing numbers of women choosing to specialize in EM over time, we evaluated changes in the proportion of female residents using simple linear regression, with the proportion of female residents serving as the outcome variable and calendar year serving as the predictor. Comparisons of independent proportions were made using the *z*-test. Data are presented as counts, proportions, and 95% confidence intervals (CI) around proportions. All *p*-values were two-tailed; we accepted *p*<0.05 as statistically significant. This study was reviewed and determined to be exempt by the Maine Medical Center Institutional Review Board.

## RESULTS

There were a total of 51,882 unique EM residents in the AMA National GME Census database during this 10-year period. The annual number of active EM residents enrolled in an ACGME-accredited program ranged from a low of 4,389 in 2006–2007 to 5,865 in 2015–2016. When comparing overall rates of attrition between EM and the other top 10 largest specialties, EM had the lowest rate of attrition (0.8%, 95% CI [0.7–0.9]), or approximately 51.6 (95% CI [44.7–58.5]) residents per year (Table 1). The majority of EM residents graduated from allopathic medical schools (82.4%, 95% CI [81.4–83.4]), followed by those from osteopathic (11.2%, 95% CI [10.5–11.9]) and international (6.4%, 95% CI [6.0–6.8]) medical schools. There were no significant differences in attrition by type of medical school graduate (χ^2^=7.150, df=2, *p*=0.028).

From 2006 to 2016, women comprised 38.8% (95% CI [37.9–39.7]) of EM residents, with no significant changes in gender composition noted during the study period (F=0.607, *p*=0.436). In the attrition population, 44.3% (95% CI [40.0–48.4]) of the residents were women, a significantly higher proportion when compared to the proportion of female EM residents overall (z=−2.544, *p*=0.011).

When examining attrition status, almost half of the residents who left their programs “withdrew” (47.0%, 95% CI [42.8–51.4]) (Table 2). There were no differences in attrition status by gender except for those who were “dismissed,” with a significantly greater proportion of men receiving this status than women (16.0% vs 8.3%; χ^2^=9.852, df=4, *p*=0.043). When examining the primary listed reason for attrition, the majority reported a “change in career plans” (57.4%, 95% CI [50.9–63.3]) (Table 3). A significantly greater proportion of women than men reported “health/family” reasons for attrition (21.5% vs 9.6%; χ^2^=9.923, df=3, *p*=0.019). All other queried reasons for attrition were similar between women and men.

Race/ethnicity responses to the AMA National GME Census were reported alone or in combination with any other race/ethnicity response. “Alone” indicated those who selected only one race/ethnicity response, whereas “in combination” indicated those who selected more than one race/ethnicity response. An individual could therefore be represented in more than one race/ethnicity category if that individual reported more than one race/ethnicity response. As such, there were 52,490 subjects in this analysis with 1.2% of the subjects reporting more than one racial/ethnic category. Whites comprised the largest group of EM residents (71.3%), followed by Asians (13.0%), Hispanics/Latinos (6.3%), Blacks/African Americans (5.0%), other (3.3%), American Indians/Alaskan Natives (0.8%), Native Hawaiians/other Pacific Islanders (0.2%), and unknown (0.1%). When comparing attrition across race/ethnicity categories, White (0.9%) residents experienced significantly less attrition than their Hispanic/Latino (1.8%) counterparts (χ^2^=32.243, df=7, *p*<0.001) (Table 4). In addition, the proportion of Hispanic/Latino residents who were “dismissed” from their programs (39.3%), was significantly greater than Asian (10.5%) and White (7.5%) residents experiencing dismissal (χ^2^=67.516, df=24, *p*<0.001) (Table 5).

## DISCUSSION

The rate of attrition for EM residents is low, and it is the lowest when compared to the other 10 largest specialties. This is consistent with results from prior work also demonstrating the relatively low rate of EM resident attrition (1.5%) compared to other specialties.^10^ Our investigation did not address whether this finding is due to a positive training environment, appropriate career selection, shorter training programs, or the resiliency of EM trainees, although all are possible contributing factors. While the low attrition rate experienced by EM programs is commendable, the premature loss of a resident during training can still be disruptive and damaging to morale for both the resident and the program. Furthermore, observations on the resident characteristics associated with attrition may inform current efforts to promote inclusion and diversity within the specialty.^12^,^13^

During the study period, we found that female EM residents were significantly more likely to leave residency than their male colleagues. Female EM residents were also less likely to be dismissed from their programs and significantly more likely to report health or family causes as the reason for their attrition during training than male residents. These findings suggest male and female EM residents may experience different demands in and outside of residency training. For example, prior work demonstrated that while depressive symptoms increased during intern year for both men and women, this increase was significantly greater for women, who cited work-family conflicts as a contributing factor.^14^ This discrepancy remains consistent among practicing emergency physicians, for whom factors associated with career satisfaction include schedule flexibility and sufficient time with family.^15^,^16^ In addition, a majority of female physicians reported deferring important life decisions (e.g., getting married, having children) in order to pursue their medical careers.^17^

While it was not clear from our data if childcare had any role in the greater likelihood of female EM residents prematurely leaving their programs, with most medical residents being of child-bearing age, it is possible that the challenges of having and raising children during training play a role in this gender discrepancy. Female residents may also be more likely to be caretakers of elderly parents or other ill family members than their male peers.^18^ Current American Board of Emergency Medicine policy on resident leave for any reason recommends up to six weeks of sanctioned time away per year without the requirement of extending residency training.^19^ However, the ABEM policy also stipulates that “if a residency program already has a policy in effect for leave time that is less than six weeks, the program may operate according to its own policy.”

While a full discussion of the history and controversies surrounding paid parental and family leave in the United States is beyond the scope of this paper,^20^ its relevance cannot be overstated in light of the increasing numbers of women who are entering medicine^21^ and the growing numbers of physicians from younger generational cohorts (e.g., millennials) who may place greater value on work-life balance than physicians from prior generations.^22^ Although our study could not discern the specific circumstances behind a resident’s choice to leave training due to “health or family reasons,” we suspect standardizing parental, family, and medical leave policies and providing affordable access and support for child and elder care may be steps to help address this gender discrepancy in attrition. Residency programs may also take creative steps to accommodate residents who need to take leave (e.g., scheduling more demanding rotations earlier in pregnancy or allowing residents to design reading or research electives that comply with Residency Review Committee-EM requirements) to minimize the time needed away from training.

There were limited racial differences in EM resident attrition. Although Asian, Hispanic/Latino, and Black/African American residents comprised greater proportions of the attrition population than the overall population, in pairwise comparisons between groups, only Hispanic/Latino residents were significantly more likely to leave and be dismissed from training than their White counterparts. It should be noted that the EM resident attrition rate in all racial/ethnic groups remained low, with each group experiencing a rate less than 2%. Nonetheless, the higher attrition rate experienced by a traditionally under-represented minority group in medicine raises questions about the unique challenges faced by physicians-in-training who are part of this under-represented group. Previous reports have noted that ethnic minority trainees perceive barriers to success in academic medicine that are based on their race.^23^–^25^ These barriers may also be present in the training environment of EM programs and could partially account for this difference in attrition.

## LIMITATIONS

There are important limitations to these results. First, this study was an investigation of broad trends and we were unable to ascribe specific causes or individual reasons contributing to a resident’s choice to leave a training program. Second, the census data relied on the report of PDs, who may have a different perspective on the reasons for attrition as compared to that of the resident. Stigma may also have caused PDs to decrease the number of residents ascribed to dismissal or withdrawal as opposed to other attrition statuses. Third, the categories of attrition statuses and reasons queried by the census were rather broad and may not encompass realities that cross multiple selections. Fourth, we were unable to obtain more granular data on resident race and ethnicity, so those who responded with two categories, for example, were double counted in analyses using race/ethnicity. However, this group of residents accounted for only 1.2% of the study population and likely had limited effects on our results. Finally, the question of what interventions could prevent resident attrition is also left unanswered, and provides fertile ground for future research.

## CONCLUSION

National rates of EM resident attrition are the lowest among similarly-sized specialties. Among EM residents who do leave their programs, women were more likely than men to leave. Women were also more likely to cite health or family reasons as the primary reason for their attrition. In addition, Hispanic/Latino residents were more likely to leave than their White counterparts. Future studies that qualitatively investigate the factors that contribute to more female and ethnic minority residents to prematurely leave their training are necessary. This work should also examine what interventions programs can take to mitigate attrition among all residents.

## Figures and Tables

**Table 1:** Mean annual resident attrition rates by medical specialty.

Specialty	Overall % (95% CI)
Anesthesiology	1.2 (1.0–1.4)
Emergency medicine	0.8 (0.7–0.9)
Family medicine	1.8 (1.5–2.1)
Internal medicine	0.9 (0.8–1.0)
Neurology	1.5 (1.2–1.8)
OBGYN	1.5 (1.2–1.8)
Pathology	1.9 (1.6–2.2)
Pediatrics	1.0 (0.8–1.2)
Psychiatry	6.0 (5.7–6.3)
Radiology	0.9 (0.8–1.0)
Surgery-general	2.7 (2.4–3.0)

*CI,* confidence interval; *OBGYN*, obstetrics and gynecology.

**Table 2:** Attrition status by EM resident gender.

Attrition status	Number of residents	Number male [%, (95% CI)	Number female [%, (95% CI)]
Dismissed	65	46 [16.0%* (12.2–20.7)]	19 [8.3%* (5.4–12.6)]
Transfer in EM	77	44 [15.3% (11.6–20.0)]	33 [14.5% (10.9–19.6)]
Transfer out of EM	63	29 [10.1% (7.12–14.1)]	34 [14.9% (10.9–20.1)]
Transfer unknown	68	33 [11.5% (8.31–15.7)]	35 [15.4% (11.3–20.6)]
Withdrawn	242	135 [47.0% (41.3–52.8)]	107 [46.9% (40.6–53.4)]
Total	515	287	228

*EM,* emergency medicine; *CI,* confidence interval; *df,* degrees of freedom.

* χ^2^=9.852; df=4; p=0.043.

**Table 3:** Attrition reason by EM resident gender.

Attrition reason	Number of residents	Number male [%, (95% CI)]	Number female [%, (95% CI)]
Change in career plans	139	81 [60.0% (51.6–67.9)]	58 [54.2% (44.8–63.3)]
Health/family reasons	36	13 [9.6%* (5.7–15.8)]	23 [21.5%* (14.8–30.2)]
Military obligations	2	0	2 [(1.9% (0.5–6.6)]
Other/unknown	65	41 [30.4% (23.2–38.9)]	24 [22.4% (15.6–31.2)]
Total	242	135	107

*EM,* emergency medicine; *CI,* confidence interval; *df,* degrees of freedom.

* χ^2^=9.923; df=3; p=0.017.

**Table 4:** EM resident race/ethnicity compositions in the overall and attrition populations.

Race/ethnicity	Total count	Total attrition count	% Overall population (95% CI)	% Attrition population (95% CI)	Attrition rate (95% CI)
White	37413	329	71.3 (67.5–75.1)	63.4 (54.1–72.6)	0.88% (0.75–1.01)
Asian	6849	76	13.0 (11.9–14.2)	14.6 (10.8–18.5)	1.11% (0.82–1.40)
Hispanic/Latino	3302	60	6.3 (5.6–7.0)	11.5 (6.0–17.1)	1.82% (0.94–2.71)
Black/African American	2613	32	5.0 (4.6–5.3)	6.2 (3.9–8.5)	1.22% (0.77–1.68)
American Indian/ Alaska Native	414	5	0.8 (0.7–0.9)	1.0 (0.1–1.8)	1.21% (0.15–2.27)
Native Hawaiian/ Pacific Islander	114	0	0.2 (0.2–0.3)	0	0
Other	1717	17	3.3 (2.9–3.7)	3.3 (1.5–5.1)	0.99% (0.45–1.53)
Unknown	68	0	0.1 (0–0.2)	0	0
Total	52490	519			

*EM,* emergency medicine; *CI,* confidence interval; *df,* degrees of freedom.

**Table 5:** Attrition status by EM resident race/ethnicity.

Attrition status	Number White [%, (95% CI)]	Number Asian [%, (95% CI)]	Number Hispanic/Latino [%, (95% CI)]	Number Black/African American [%, (95% CI)]
Dismissed	22 [7.4%* (5.0–11.0)]	8 [10.5%* (5.4–19.4)]	22 [39.3%* (27.6–52.4)]	3 [9.4%* (3.2–24.2)]
Transfer in EM	43 [14.6% (11.0–19.1)]	14 [18.4% (11.3–28.6)]	11 [19.6% (11.3–31.8)]	3 [9.4% (3.2–24.2)]
Transfer out of EM	42 [14.2% (10.7–18.7)]	12 [15.8% (9.3–25.6)]	2 [3.6% (0.98–12.1)]	5 [15.6% (6.9–31.8)]
Transfer unknown	43 [14.6% (11.0–19.1)]	13 [17.1% (10.3–27.1)]	3 [5.4% (1.8–14.6)]	3 [9.4% (3.2–24.2)]
Withdrawn	145 [49.2% (43.5–54.8)]	29 [38.2% (28.1–49.4)]	18 [32.1% (21.4–45.2)]	18 [56.2% (39.3–71.8)]
Total	295	76	56	32

*EM,* emergency medicine; *CI,* confidence interval; *df,* degrees of freedom.

* χ^2^=67.516; df=24; p<0.001.

American Indian/Alaska Native, Native Hawaiian/Pacific Islander, Other, and Unknown were not included due to their small sample sizes.
